# Clustering Heart Rate Dynamics Is Associated with β-Adrenergic Receptor Polymorphisms: Analysis by Information-Based Similarity Index

**DOI:** 10.1371/journal.pone.0019232

**Published:** 2011-05-04

**Authors:** Albert C. Yang, Shih-Jen Tsai, Chen-Jee Hong, Cynthia Wang, Tai-Jui Chen, Ying-Jay Liou, Chung-Kang Peng

**Affiliations:** 1 Department of Psychiatry, Chu-Tung Veterans Hospital, Hsin-Chu County, Taiwan; 2 Center for Dynamical Biomarkers and Translational Medicine, National Central University, Chungli, Taiwan; 3 Division of Psychiatry, School of Medicine, National Yang-Ming University, Taipei, Taiwan; 4 Department of Psychiatry, Taipei Veterans General Hospital, Taipei, Taiwan; 5 Institute of Brain Science, National Yang-Ming University, Taipei, Taiwan; 6 Department of Molecular Biology, Princeton University, Princeton, New Jersey, United States of America; 7 I-Shou University and E-DA Hospital, Kaohsiung, Taiwan; 8 Margret and H. A. Rey Institute for Nonlinear Dynamics in Medicine, Beth Israel Deaconess Medical Center/Harvard Medical School, Boston, Massachusetts, United States of America; Istituto Dermopatico dell'Immacolata, Italy

## Abstract

**Background:**

Genetic polymorphisms in the gene encoding the β-adrenergic receptors (β-AR) have a pivotal role in the functions of the autonomic nervous system. Using heart rate variability (HRV) as an indicator of autonomic function, we present a bottom-up genotype–phenotype analysis to investigate the association between β-AR gene polymorphisms and heart rate dynamics.

**Methods:**

A total of 221 healthy Han Chinese adults (59 males and 162 females, aged 33.6±10.8 years, range 19 to 63 years) were recruited and genotyped for three common β-AR polymorphisms: β_1_-AR Ser49Gly, β_2_-AR Arg16Gly and β_2_-AR Gln27Glu. Each subject underwent two hours of electrocardiogram monitoring at rest. We applied an information-based similarity (IBS) index to measure the pairwise dissimilarity of heart rate dynamics among study subjects.

**Results:**

With the aid of agglomerative hierarchical cluster analysis, we categorized subjects into major clusters, which were found to have significantly different distributions of β_2_-AR Arg16Gly genotype. Furthermore, the non-randomness index, a nonlinear HRV measure derived from the IBS method, was significantly lower in Arg16 homozygotes than in Gly16 carriers. The non-randomness index was negatively correlated with parasympathetic-related HRV variables and positively correlated with those HRV indices reflecting a sympathovagal shift toward sympathetic activity.

**Conclusions:**

We demonstrate a bottom-up categorization approach combining the IBS method and hierarchical cluster analysis to detect subgroups of subjects with HRV phenotypes associated with β-AR polymorphisms. Our results provide evidence that β_2_-AR polymorphisms are significantly associated with the acceleration/deceleration pattern of heart rate oscillation, reflecting the underlying mode of autonomic nervous system control.

## Introduction

Instantaneous heart rate in response to physiological perturbations often exhibits remarkable oscillations at multiple time scales. These oscillations, known as heart rate variability (HRV), are mainly mediated by the autonomic nervous system via parasympathetic and sympathetic innervations. Analysis of HRV has been suggested to reveal subtle patterns of heart rate dynamics that are relevant to the underlying physiological state and autonomic nervous system function [1]. Prior studies have shown that HRV measures are highly heritable traits that can be used to support genetic association and linkage studies [2], [3], [4], [5]. Family and twin studies have shown a significant genetic influence on a variety of HRV measures [6], [7]. Genetic polymorphisms related to cardiovascular functions have been associated with altered HRV [8], [9], [10], [11]. Recently, we have also made progress in identifying variations in two genes related to neuropsychiatric function that are associated with altered heart rate dynamics in samples of healthy adult and elderly subjects: those encoding brain-derived neurotrophic factor (BDNF) [12] and apolipoprotein E [13], respectively.

Despite increasing focus on investigating the genetic influence on autonomic functions, current approaches to genotype–HRV associations have largely been characterized by a top-down approach involving a direct comparison of continuous HRV variables among pre-defined groups of subjects (i.e., healthy vs. ill or groups of known genotypes), yet it is unclear how a particular genetic polymorphism may determine a similar pattern of autonomic heart rate control from one subject to another. Specifically, heart rate dynamics is a phenotypic “expression” of the autonomic nervous system, so comparing similar heart rate oscillation phenotypes among individuals may reveal a global profile of autonomic function relevant to genetic variants. With these considerations in mind, in the present study, we introduce a bottom-up genotype–phenotype analysis to investigate the association between genetic polymorphisms and autonomic control of heart rate dynamics, using three common polymorphisms in genes encoding β-adrenergic receptor (β-*AR*) as an example.

β-*AR* has a pivotal role in the functions of the cardiac autonomic nervous system. Activation of β_1_-AR provides strong stimulus to increase the frequency and contractility of the heart, whereas the activation of β_2_-AR results in smooth muscle relaxation and increased cardiac output with less extent compared to β_1_-AR. Thus, the connection between β-*AR* and HRV is plausible and warrants evaluations. Limited evidences suggest that variations in genes coding subtypes of β-AR may be associated with heart rate or HRV. For example, Ser49Gly polymorphism in β_1_-AR gene has been found to be associated with resting heart rate [14], [15], and an association of β_2_-AR gene polymorphisms with spectral components of HRV measures has been reported in a relatively small healthy adult male sample [11].

We applied an information-based similarity index (IBS) [16], [17] to measure the pairwise dissimilarity of interbeat interval time series among a sample of healthy adult volunteers. The IBS method is based on rank-order frequency analysis of acceleration/deceleration patterns of heart rate fluctuation. Because stimulus of β-*AR* results in acceleration of heart rate, the functional changes in genetic polymorphisms of β-*AR* may affect acceleration/deceleration patterns of heart rate, which can be detected by the IBS method. The analyses of the present study were two-fold: 1) a non-randomness index [17] derived from the IBS method was applied to quantify the nonlinear aspect of HRV according to β-*AR* genotype and to test the correlation of this index with standard HRV indices; and 2) using agglomerative hierarchical cluster analysis, we unsupervisedly categorized these subjects into clusters based on pairwise dissimilarity among heart rate dynamics, and then we investigated the association of these clustering patterns with β-*AR* gene polymorphisms. We show that this bottom-up, categorization approach combining the IBS method and hierarchical cluster analysis can detect subgroups of subjects based on phenotypes that are associated with β-*AR* gene polymorphisms.

## Materials and Methods

### Subjects

Two hundred forty-seven healthy Han Chinese adult volunteers were recruited from two medical centers: Taipei Veterans General Hospital and Kaohsiung E-DA Hospital, Taiwan. Subjects were recruited by advertisement among medical employees, research laboratory staff working at both hospitals, and their relatives. All subjects gave informed consent before commencement of the study. The protocol was approved by the institutional review boards of the Taipei Veterans General Hospital (Taipei, Taiwan) and E-DA Hospital (Kaohsiung, Taiwan). Each subject was given an interview using a standard questionnaire to carefully review the history of medical disease, psychiatric illness, and medication use. Subjects included in the study did not have a personal history of medical conditions (e.g., malignant tumors, heart failure, or diabetes mellitus), pregnancy, psychiatric illnesses or substance abuse/dependence. None of the subjects was taking any medication. The collected demographic data included age, sex, body mass index, and smoking. Of note, most volunteers were hospital colleagues, and the rate of smoking was low (n = 2, 0.9%).

Of these subjects, 228 were successfully contacted for ambulatory electrocardiogram (ECG) monitoring. Holter recordings (MyECG E3-80 Portable Recorder, Microstar Inc., Taipei, Taiwan) were used to obtain two hours of ECG signals. The E3-80 device continuously recorded three channels of ECG signals at a sampling rate of 250 Hz. All ECG monitoring took place in the daytime, and participants were asked to avoid smoking or drinking alcoholic beverages and to stay in a resting state while being monitored. Valid DNA samples were obtained in 221 subjects by drawing blood or by buccal swabs. The final study sample therefore consisted of 221 healthy adult subjects (59 males and 162 females, aged 33.6±10.8 years, range 19–63 years). Among the present study sample, 211 subjects have been included elsewhere in a previous report on the altered sympathovagal balance associated with Val66Met polymorphisms of the *BDNF* gene [12].

### Genotyping

Each subject was genotyped for three polymorphisms (rs1801252, rs1042713 and rs1042714), and genomic DNA was isolated using the PUREGENE DNA purification system (Gentra Systems, Minneapolis, MN, USA). The genotypes of rs1042713 were determined using polymerase chain reaction and restriction fragment length polymorphism analysis. Briefly, primers and probes were designed with SpectroDESIGNER software (Sequenom, San Diego, CA, USA). PCR was then performed, and unincorporated double-stranded nucleotide triphosphate bases (dNTPs) were dephosphorylated with shrimp alkaline phosphatase (Hoffman-LaRoche, Basel, Switzerland) followed by primer extension. The purified primer extension reaction product was spotted on to a 384-element silicon chip (SpectroCHIP, Sequenom) and analyzed in a Bruker Biflex III MALDI-TOF SpectroREADER mass spectrometer (Sequenom). The resulting spectra were then processed with SpectroTYPER (Sequenom). All samples were genotyped for eight unrelated SNPs for DNA quality examination. The samples were diluted onto 96-well plates, and only the plates on which each of the eight unrelated SNPs had a successful genotyping rate greater than 95% were used for further study. All experiments were performed by investigators who were blind to phenotype. Failure in genotyping for rs1801252 polymorphism was noted in 6 cases.

### Analysis of heart rate variability

The ECG signals were automatically processed and analyzed by open-source HRV algorithms [18]. The standard HRV analysis has been well reviewed [19]. Briefly, time domain measures of HRV include the mean heart rate and standard deviation of the normal interbeat intervals (SDNN), the root mean square successive difference between adjacent normal interbeat intervals (RMSSD), and the percentage of adjacent intervals that varied by greater than 50 ms (pNN50) [20]. The SDNN assesses the overall variability of interbeat intervals. The RMSSD and pNN50 measure the short-term variation of interbeat intervals, which is mainly modulated by parasympathetic innervation [21]. Standard spectral HRV measures [19] include high-frequency power (HF; 0.15–0.40 Hz), low-frequency power (LF; 0.04–0.15 Hz), and very low-frequency power (VLF; 0.003–0.04 Hz). LF power is suggested to be modulated by both sympathetic and parasympathetic activities, whereas HF power is mainly modulated by parasympathetic activity [22], [23]. The LF/HF ratio is considered a measure of the shift of sympathovagal balance toward sympathetic activity [19], [24]. The physiological mechanism underlying VLF power is disputed but has been suggested to be mediated partly by the renin–angiotensin–aldosterone system or parasympathetic modulation [19], [25], [26].

In addition, we incorporated two nonlinear HRV indices: detrended fluctuation analysis (DFA) [27], [28] and multiscale entropy (MSE) [29]. DFA quantifies the presence of long-range (fractal) correlations whereas MSE measures the entropy over multiple time scales inherent in physiologic signals and is therefore a complexity measure. Both methods are available at Physionet (http://physionet.org), a research resource for complex physiologic signals [18].

In the DFA method, the root-mean-square fluctuation of integrated and detrended time series is measured at different observation windows and plotted against the size of the observation window on a log-log scale. The scaling exponent α is then derived from the slope of line fitting to the obtained log-log plot. The short-term exponent α_1_ (4 to 11 heartbeats) and the long-term scaling exponents α_2_ (>11 heartbeats) were also calculated [27], [30], [31]. Low-exponent values represent reduced fractal propertiy of heart rate dynamics and have been implicated in the risk of fatal cardiac arrhythmia, increased mortality, or poor prognosis in cardiovascular diseases [32], [33], [34], [35].

MSE has been proposed as a biologically meaningful complexity measure by quantifying the entropy over multiple time scales inherent in physiologic signals. The procedure and calculation of the MSE is summerized as following three steps: 1) construction of coarse-grained time series, 2) quantification of the sample entropy of each coarse-grained time series, and 3) summation of the sample entropy values over a range of scales. In the present study, sample entropy was calculated using a pattern length (m) of 2 and a similarity factor (r) of 0.15. The sum of sample entropy over all scale factors from 1 to 20 was computed to represent the overall MSE measure. In addition, the sum of sample entropy over scale factors from 1–5 and 6–20 was calculated to represent short-term and long-term MSE measures, respectively [36].

### Analysis of information-based similarity index

Several methods of symbolic dynamic analysis of HRV have been proposed previously [37], [38], [39], [40], [41]. The IBS method was developed to effectively categorize symbolic sequences according to their information content. The method has been fully described and validated [16], with applications to heart rate time series, literary texts, and genetic sequences [17], [42], [43].

**Figure 1:**
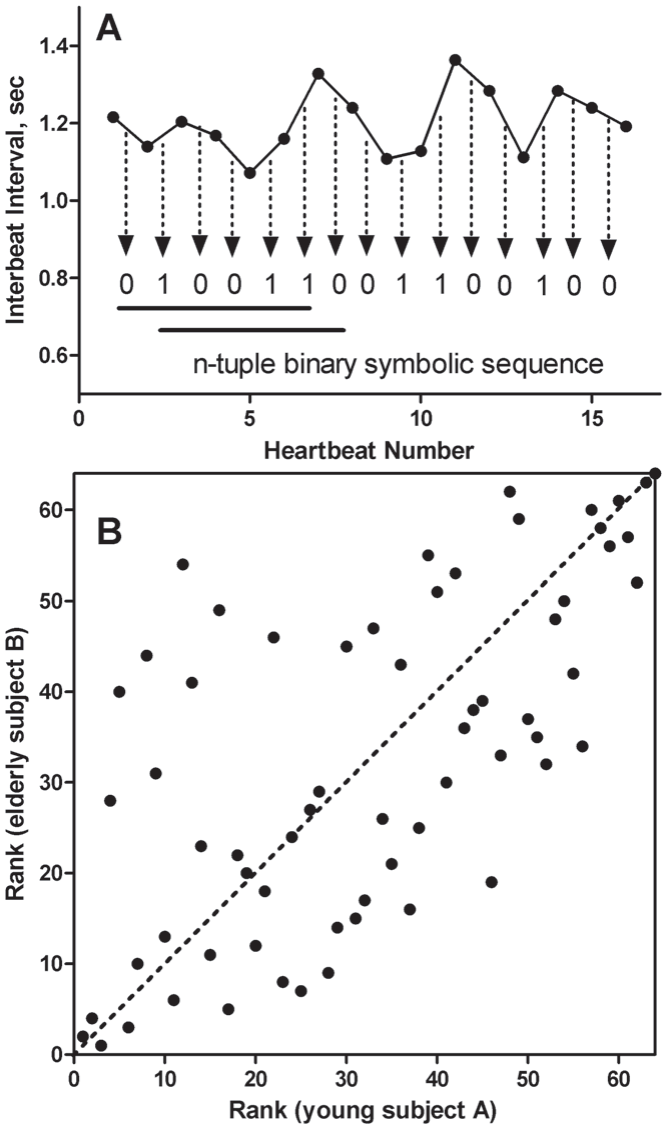
Schematic illustration of the information-based similarity index method. (A) The mapping procedure for a n-tuple binary symbolic sequence (here n = 6 for illustrative purposes) from part of an interbeat interval time series. (B) Rank-order comparison of two interbeat interval time series from the two healthy subjects, using 6-tuple binary symbolic mapping. In this case, frequencies of 2^6^ = 64 6-tuple symbolic patterns were determined and ranked accordingly. For each 6-tuple symbolic sequence (black dot), its rank in subject A is plotted against its rank in subject B. The dashed diagonal line indicates the case where the rank order of 6-tuple symbolic sequence for both subjects is identical.

An interbeat interval time series (or heart rate time series) is mapped to a symbolic sequence, according to a mapping rule that accelerated heart rate in consecutive heartbeats is designated as *0* and a deceleration of heart rate is designated as *1* (Figure 1A). This way of mapping captures the essential dynamics of the autonomic nervous system's control of heart rate and is less sensitive to noisy fluctuations in interbeat interval time series commonly caused by ectopic heartbeats [17]. A binary, symbolic “word” is then defined as a n-tuple sequence derived from n+1 consecutive interbeat intervals. We determined the frequencies of each pattern of n-tuple sequences by applying a sliding window (moving one interbeat interval/step) across the entire interbeat interval time series and then ranked each n-tuple sequence according to its frequency in descending order.

To compare the similarity between symbolic sequences, we plotted the rank number of each n-tuple sequence in the first sequence against that of the second sequence (Figure 1B). If two sequences are similar in their rank order of n-tuples, the scattered points will be located near the diagonal line (e.g., comparison between healthy subjects) [17]. Therefore, the average deviation of these scattered points away from the diagonal line is a measure of the dissimilarity index between these two sequences.

We defined the distance 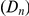 using *n*-tuples between two sequences, 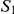 and 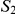, as
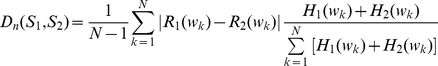
Here 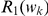 and 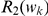 represent the rank of a specific n-tuple, 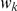, in sequences 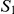 and 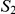, respectively. N = 2*^n^* is the number of different n-tuple sequences (or patterns). The absolute difference of ranks, 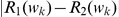, is weighted by summing Shannon's entropy *H* for 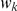 in sequences 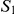 and 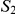 [44]. Shannon's entropy measures the information richness of each n-tuple in both sequences. Thus, the more frequently used n-tuples contribute more to measuring similarity among symbolic sequences. Of note, the IBS is an empirical index which does not necessarily obey the triangular inequality criterion of a distance measure. Therefore, the triangular inequality test is required before generating a cluster [16], [17]. The IBS algorithm was available at Physionet (http://www.physionet.org/physiotools/ibs/).

The applications of the IBS method in the present study were two-fold: 1) The pairwise distance of heart rate dynamics among individuals was calculated by an average of IBS distance using n-tuple symbolic sequences (n = 2–10) and was used in subsequent hierarchical cluster analysis. 2) To quantify the nonlinear aspect of heart rate time series, a non-randomness index was defined to measure the symbolic patterns of heart rate dynamics away from complete randomness (i.e., absence of ordered control of the autonomic nervous system) [17]. The non-randomness index is independent of conventional entropic measures (i.e., sample entropy or approximate entropy) and has been evaluated in other studies [17], [45], [46], [47]. The calculation of the non-randomness index is based on estimating the average n-tuple distance between raw interbeat interval time series and its randomly shuffled surrogates using the IBS method, where the number of surrogates was 100 in the present study. The averaged non-randomness index derived from each n-tuple non-randomness index (n = 2–10) was then used in this study for comparisons among β-*AR* genotypes.

### Statistical analysis

We calculated allele and genotype frequencies and performed Hardy–Weinberg equilibrium tests for each β-*AR* genotype. The spectral HRV indices were log-transformed to produce normalized distributions. To control for the effects of non-genetic factors, differences in HRV variables were compared for individual genotypes using a general linear model (GLM) followed by the Bonferroni post hoc test for corrections of multiple between-group comparisons., using age, gender, and body mass index as covariates. Effect sizes were calculated with Cohen *d*, defined as the difference between the means of two groups, divided by the pooled standard deviation of these groups. Partial correlation analysis was applied, controlling for age and BMI, to determine the associations between standard HRV indices and non-randomness index derived from the IBS method. A p value of less than 0.05 (two-tailed) was required for all statistical comparisons.

A complete-linkage, hierarchical clustering tree was estimated using the generalized association plot (GAP) (http://gap.stat.sinica.edu.tw/). GAP is implemented here as an unsupervised clustering algorithm to visually categorize all subjects based on the dissimilarity matrix, which is derived from calculating the pairwise dissimilarity of heart rate dynamics among subjects using the IBS method. Cluster-specific association was analyzed by the chi-square test to assess the frequency of β-*AR* polymorphisms and by the GLM model to test the differences in standard HRV indices among resulting clusters. To validate cluster-specific associations, we tested the differences in HRV and β-*AR* genotypes in estimated clusters based on first- and second-half ECG data.

## Results

### Demographic data

Demographic and clinical data for subjects with each β-*AR* genotype are presented in Table S1 and S2. The subgroups in three genotypes did not differ in age, gender ratio, smoking status, or body mass index. There was no detectable deviation from Hardy–Weinberg equilibrium in β_1_-*AR* Ser49Gly genotype (*χ^2^* = 0.169, *P* = 0.681), β_2_-*AR* Arg16Gly genotype (*χ^2^* = 0.824, *P* = 0.364) or β_2_-*AR* Gln27Glu genotype (*χ^2^* = 2.188, *P* = 0.139).

### Association of β-AR genotypes with standard heart rate variability

Tables 1, 2, 3 summarize the association of β-*AR* genotypes with HRV indices determined by a GLM model using age, gender, and BMI as covariates. There was no significant effect of interaction on HRV indices detected between β-*AR* genotypes and demographic variables.

**Table 1:** Heart rate variability profile according to β1-adrenergic receptor Ser49Gly genotype.

	Ser/Sern = 155	Gly allelen = 60	*p*
**Time domain**			
Mean heart rate, beats/minute	82.9±13.2	86.2±12.0	0.087
SDNN, ms	77.3±24.4	70.7±20.2	0.064
RMSSD, ms	30.6±13.5	28.0±13.3	0.204
pNN50, %	10.4±11.0	9.1±10.5	0.448
**Frequency domain**			
VLF power, ln(ms^2^/Hz)	8.56±0.53	8.46±0.61	0.527
LF power, ln(ms^2^/Hz)	7.37±0.62	7.32±0.76	0.580
LF percentage, %	22.0±6.4	23.0±7.0	0.345
HF power, ln(ms^2^/Hz)	6.47±0.84	6.33±0.96	0.293
HF percentage, %	10.0±6.1	9.3±5.1	0.488
LF/HF ratio, normalized units	3.16±1.48	3.50±1.94	0.170
**Nonlinear domain**			
Detrended fluctuation analysis, α	0.90±0.07	0.90±0.07	0.911
Detrended fluctuation analysis, α_1_	1.20±0.18	1.24±0.19	0.199
Detrended fluctuation analysis, α_2_	1.07±0.09	1.06±0.08	0.493
Multiscale entropy, scale 1–20	28.8±5.75	29.56±5.74	0.397
Multiscale entropy, scale 1–5	6.6±1.5	6.7±1.6	0.657
Multiscale entropy, scale 6–20	22.2±4.4	22.8±4.4	0.340
Non-randomness, symbolic word length 2–10	0.32±0.11	0.33±0.11	0.348

Failure in genotyping for β1-AR Ser49Gly polymorphism was noted in 6 cases.

**Table 2:** Heart rate variability profile according to β2-adrenergic receptor Gln27Glu genotype.

	Gln/Glnn = 181	Glu allelen = 40	*p*
**Time domain**			
Mean heart rate, beats/minute	83.8±13.0	84.0±12.8	0.290
SDNN, ms	76.9±24.6	71.7±18.1	0.207
RMSSD, ms	30.2±13.7	29.3±13.3	0.698
pNN50, %	10.2±11.1	10.3±10.9	0.987
**Frequency domain**			
VLF power, ln(ms^2^/Hz)	8.54±0.58	8.50±0.45	0.674
LF power, ln(ms^2^/Hz)	7.36±0.68	7.34±0.63	0.845
LF percentage, %	22.2±6.6	22.5±6.5	0.763
HF power, ln(ms^2^/Hz)	6.45±0.89	6.39±0.90	0.728
HF percentage, %	9.8±6.0	9.8±5.5	0.763
LF/HF ratio, normalized units	3.26±1.65	3.23±1.62	0.973
**Nonlinear domain**			
Detrended fluctuation analysis, α	0.90±0.07	0.90±0.07	0.940
Detrended fluctuation analysis, α_1_	1.21±0.19	1.22±0.18	0.749
Detrended fluctuation analysis, α_2_	1.07±0.09	1.07±0.09	0.868
Multiscale entropy, scale 1–20	28.7±5.9	29.8±5.2	0.283
Multiscale entropy, scale 1–5	6.6±1.5	6.9±1.6	0.288
Multiscale entropy, scale 6–20	22.1±4.5	22.9±3.9	0.302
Non-randomness, symbolic word length 2–10	0.32±0.11	0.31±0.11	0.793

**Table 3:** Heart rate variability profile according to β2-adrenergic receptor Arg16Gly genotype.

	Arg16/Arg16n = 78	Arg16/Gly16n = 101	Gly16/Gly16n = 42	*P*(codominant)	*P*(Gly-dominant)
**Time domain**					
Mean heart rate, beats/minute	83.7±14.5	83.8±12.0	84.1±12.1	0.991	0.947
SDNN, ms	77.4±24.9	75.4±24.8	74.3±17.9	0.756	0.484
RMSSD, ms	31.0±14.5	30.1±13.3	28.5±12.7	0.633	0.470
pNN50, %	11.1±12.4	9.9±10.3	9.6±10.3	0.711	0.416
**Frequency domain**					
VLF power, ln(ms^2^/Hz)	8.54±0.65	8.54±0.51	8.52±0.47	0.967	0.891
LF power, ln(ms^2^/Hz)	7.31±0.74	7.43±0.63	7.27±0.62	0.284	0.423
LF percentage, %	21.2±5.9	23.5±6.9	21.2±6.4	0.028	0.069
HF power, ln(ms^2^/Hz)	6.42±0.97	6.50±0.82	6.33±0.90	0.575	0.818
HF percentage, %	10.0±7.2	10.0±5.3	9.1±4.6	0.702	0.784
LF/HF ratio, normalized units	3.17±1.73	3.28±1.63	3.35±1.52	0.830	0.375
**Nonlinear domain**					
Detrended fluctuation analysis, α	0.90±0.07	0.90±0.06	0.91±0.07	0.237	0.817
Detrended fluctuation analysis, α_1_	1.19±0.19	1.23±0.19	1.20±0.16	0.237	0.189
Detrended fluctuation analysis, α_2_	1.07±0.09	1.06±0.09	1.09±0.09	0.222	0.658
Multiscale entropy, scale 1–20	28.5±5.6	29.5±5.8	28.2±6.0	0.363	0.488
Multiscale entropy, scale 1–5	6.5±1.5	6.8±1.6	6.4±1.7	0.287	0.558
Multiscale entropy, scale 6–20	22.0±4.3	22.7±4.4	21.8±4.5	0.421	0.482
Non-randomness, symbolic word length 2–10	0.29±0.11	0.34±0.11	0.33±0.09	0.004	0.001

β_1_-*AR* Ser49Gly genotype was associated with mean heart rate and SDNN with borderline significance (Table 1; p = 0.087 and p = 0.064, respectively). No statistical association was found between β_2_-*AR* Gln27Glu genotype and HRV indices (Table 2). For the β_2_-*AR* Arg16Gly genotype (Table 3), a significant codominant association was found with LF% (F = 3.636, p = 0.028) and the non-randomness index derived from the IBS method (F = 5.642, p = 0.004). Multiple comparisons showed that LF% was significantly lower in homozygous Arg16 carriers compared to subjects with the Arg16/Gly16 genotype (p = 0.016), but not to homozygous Gly16 carriers (p = 0.985). Likewise, a significantly lower non-randomness index was found in homozygous carriers of the Arg16 allele (p = 0.016) compared to subjects heterozygous for Arg16/Gly16 (p = 0.001), and a borderline significance was seen in the comparison to homozygous Gly16 carriers (p = 0.057).

There was no significant difference in HRV indices between heterozygotes Arg16/Gly16 and homozygous Gly16 carriers, indicating a dominant association of the Gly16 allele with HRV indices. Thus, when the sample was stratified according to the presence of the Gly16 allele, a significantly lower non-randomness index was detected in Arg16 homozygotes compared to subjects with one or two Gly16 alleles (p = 0.001). The effect size of the non-randomness index among Gly16 and non-Gly16 groups was 0.22 (Cohen *d* = 0.46). The Gly16 and non-Gly16 carriers trended toward a significant difference in LF% (p = 0.069).

### Correlations between non-randomness index and standard heart rate variability

To ascertain the relationship of non-randomness index with autonomic function, we estimated the partial correlation between non-randomness index and standard HRV variables, controlling for age and BMI (Table S3). A weak but significant positive correlation of the non-randomness index with the LF/HF ratio was found (r = 0.234, p = 0.001), whereas negative correlations were found with RMSSD (r = −0.274, p<0.001) and pNN50 (r = −0.279, p<0.001).

### Association of heart rate dynamic clusters with β-AR gene polymorphisms

**Figure 2:**
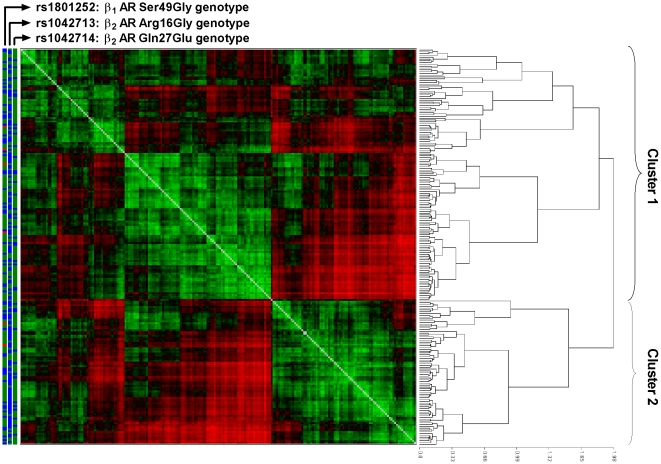
Unsupervised, hierarchical cluster tree of subjects according to the pairwise dissimilarity matrix among heart rate dynamics using an information-based similarity index method. Dissimilarity matrix data were visualized and clustered by a generalized association plot algorithm [58]. The bars on the left indicate the β-adrenergic receptor (β-AR) gene polymorphisms rs1801252 (green: homozygous Ser49 carriers; blue: Gly16 allele carriers; red: unknown genotype), rs1042713 (green: homozygous Arg16 carriers; blue: Gly16 allele carriers) and rs1042714 (green: homozygous Gln27 carriers; blue: Glu27 allele carriers).

Next, we investigated the β-*AR* genotype distributions in an unsupervised cluster tree based on the dissimilarity (distance) measure of heart rate dynamics. We first estimated the pairwise distance between interbeat interval time series of all subjects using the IBS method. No violation of the triangular inequality was observed. We then applied a hierarchical clustering algorithm to cluster these subjects based on the obtained distance matrix. Two major clusters were identified in the resulting dendrogram (Figure 2). There was no significant difference in age, gender, or other demographic variables between the two clusters (data not shown). Table 4 shows β-*AR* genotypes and standard HRV characteristics according to two major clusters. A significant deviation in the distribution of homozygous β_2_-*AR* Arg16 carriers was detected between the two clusters (p = 0.011), whereas the other genotypes showed no deviation in genotype distribution.

**Table 4:** Genotype and standard heart rate variability characteristics according to two major clusters by a generalized association plot.

	Cluster 1n = 141	Cluster 2n = 80	*p*
β1-AR Ser49Gly, n (Ser/Ser vs. Gly allele)*	36/101	24/54	0.584
β2-AR Arg16Gly, n (Arg/Arg vs. Gly allele)	59/82	19/61	0.011
β2-AR Gln27Glu, n (Gln/Gln vs. Gln/Glu)	115/26	66/14	1.000
**Standard heart rate variability**			
Mean heart rate, beats/minute	84.8±14.4	82.0±9.6	0.653
SDNN, ms	76.4±25.9	75.1±19.0	0.656
RMSSD, ms	29.9±14.3	30.4±12.4	0.788
pNN50, %	10.2±11.5	10.3±10.3	0.930
VLF power, ln(ms^2^/Hz)	8.50±0.61	8.61±0.44	0.117
LF power, ln(ms^2^/Hz)	7.21±0.69	7.62±0.55	<0.001
HF power, ln(ms^2^/Hz)	6.68±0.73	6.30±0.94	0.001
LF/HF ratio, normalized units	3.29±1.71	3.19±1.52	0.670

AR: adrenergic receptor.

*Failure in genotyping for β1-AR Ser49Gly polymorphism was noted in 6 cases.

In terms of standard HRV characteristics, cluster 1 (with a higher rate of homozygous β_2_-*AR* Arg16 carriers) showed significantly reduced LF power (p<0.001) and significantly increased HF power (p = 0.001). The clusters did not differ in other HRV indices. We verified the genotype distribution with clusters based on first- and second-half ECG data, and the results were consistent (Table S4).

## Discussion

The data presented in this study demonstrate a significant association of a common β_2_-*AR* polymorphism, Arg16Gly, with the non-randomness index, a nonlinear HRV measure derived from the IBS method. Moreover, we illustrate that a bottom-up approach using the IBS method was able to measure the dissimilarity of heart rate dynamics among individuals, and we show that the resulting clusters were associated with β_2_-*AR* Arg16Gly genotype, indicating an impact of this β_2_-*AR* polymorphism on acceleration/deceleration patterns of heart rate oscillations.

Our study offers several advantages for studying genetic associations with physiological parameters and might be generalizable to other genotype–phenotype studies based on different types of time series (e.g., brain electroactivity or time series derived from functional magnetic resonance imaging). First, our method enables us to cluster heart rate dynamics by an IBS method based on their acceleration/deceleration patterns of heartbeat sequence. The IBS method was not exclusively developed for the analysis of heartbeat sequence but also for other generic datasets consisting of repetitive elements [42], [43]. With an appropriate mapping rule that is meaningful to a target dataset, the IBS method can be applied to other types of phenotypic data derived from the time series [16]. Second, we employed a visually aided hierarchical analysis by a software tool called a generalized association map, which enables a bottom-up approach to unsupervisedly identify heart rate clusters to study genotype–phenotype associations. This bottom-up approach may help reduce the false-positivity commonly seen in conventional association studies.

β_1_-*AR* is the predominant β-*AR* subtype in the heart. Compared to activation of β_2_-*AR*, stimulation in β_1_-*AR* can result in more significantly increased frequency and contractility of heart. Although substantial efforts have been made to link β_1_-*AR* polymorphisms with cardiovascular function, studies have been mixed in this regard. For example, β_1_-*AR* Ser49Gly polymorphism was found to be associated with increased blood pressure and heart rate [14], [15] but data are inconsistent in our findings and other reports [48], [49]. Because we studied only one polymorphism in β_1_-*AR*, further research is needed to identify other possible β_1_-*AR* polymorphisms associated with HRV.

This study found that β_2_-*AR* Arg16Gly genotype was associated with clusters based on acceleration/deceleration patterns of heart rate. Although β_2_-*AR* is expressed in the heart at lower concentrations than is the β_1_-*AR* subtype, it also expresses on the presynaptic sympathetic nerve terminals and expresses abundantly in vascular and bronchial smooth muscle. Therefore, changes in β_2_-*AR* function may not only alter sympathetic activity [50], but also respiration and vascular responses [51], [52]. It can be reasonable to speculate that these factors have influences in HRV. Of note, the association between β_2_-*AR* Arg16Gly polymorphism and heart rate fluctuation patterns may simply reflect a functional genetic component nearby due to linkage disequilibrium and warrants future genetic research targeting on this region.

The non-randomness index was developed initially as a nonlinear HRV index [17] and its connection with other traditional HRV indices needs to be explored. Though the non-randomness index was weakly associated with HRV indices (r-square<0.1; Table S3), it did correlate negatively with HF power and positively with the LF/HF ratio, suggesting that the non-randomness index is a marker related to sympathovagal balance. Our findings of lower non-randomness index and LF% seen in homozygous β_2_-*AR* Arg16 carriers are in line with prior studies showing that the β_2_-*AR* Arg16 polymorphism is associated with lower sympathetic activity, manifested as lower blood pressure [53], [54], lower plasma norepinephrine [54], [55], and enhanced agonist-mediated desensitization in the vasculature [56].

Strength of this work include a larger sample size, matched gender distribution, and long recording of ECG signals using a Holter recorder, compared to prior genetic association study of HRV. Several limitations influence the interpretation of the findings presented in this study. First, we are unable to repeat findings of spectral HRV indices seen in a smaller male sample [11]. This may be due to different gender distributions [57] and means of ECG recording. Twenty-four hour Holter recording could validate and reinforce the association of β-*AR* genetic polymorphism with HRV seen in this study. Second, we were unable to evaluate the correlation of the non-randomness index with other commonly used sympathetic indicators, such as blood pressure or plasma catecholamine levels. Third, our findings are based on a healthy population and may not be generalizable to medically ill patients, such as those with cardiovascular diseases. The cross-sectional nature of our study also limited us examining the age modification of the effect of β-*AR* receptor polymorphism on autonomic function.

In conclusion, this study shows that a bottom-up, categorization approach combining the IBS method and hierarchical cluster analysis can detect subgroups of subjects based on phenotypes that are associated with β_2_-*AR* gene polymorphisms. Further research should aim to identify the physiological mechanisms underlying these findings.

## Supporting Information

Table S1 Demographic data according to β1-adrenergic receptor Ser49Gly genotype.
(DOC)

Table S2 Demographic data according to β2-adrenergic receptor Arg16Gly and Gln27Glu genotype.
(DOC)

Table S3 Partial correlations between non-randomness index and heart rate variability components.
(DOC)

Table S4 Genotype and standard heart rate variability characteristics according to two major clusters by a generalized association plot.
(DOC)

## References

[1] Goldberger AL, Amaral LA, Hausdorff JM, Ivanov P, Peng CK (2002). Fractal dynamics in physiology: alterations with disease and aging.. Proc Natl Acad Sci U S A.

[2] Singh JP, Larson MG, O'Donnell CJ, Tsuji H, Evans JC (1999). Heritability of heart rate variability: the Framingham Heart Study.. Circulation.

[3] Kupper NH, Willemsen G, van den Berg M, de Boer D, Posthuma D (2004). Heritability of ambulatory heart rate variability.. Circulation.

[4] Wang X, Thayer JF, Treiber F, Snieder H (2005). Ethnic differences and heritability of heart rate variability in African- and European American youth.. Am J Cardiol.

[5] Newton-Cheh C, Guo CY, Wang TJ, O'Donnell CJ, Levy D (2007). Genome-wide association study of electrocardiographic and heart rate variability traits: the Framingham Heart Study.. BMC Med Genet.

[6] Sinnreich R, Friedlander Y, Luria MH, Sapoznikov D, Kark JD (1999). Inheritance of heart rate variability: the kibbutzim family study.. Hum Genet.

[7] Su S, Lampert R, Zhao J, Bremner JD, Miller A (2009). Pleiotropy of C-reactive protein gene polymorphisms with C-reactive protein levels and heart rate variability in healthy male twins.. Am J Cardiol.

[8] Busjahn A, Voss A, Knoblauch H, Knoblauch M, Jeschke E (1998). Angiotensin-converting enzyme and angiotensinogen gene polymorphisms and heart rate variability in twins.. Am J Cardiol.

[9] Neumann SA, Lawrence EC, Jennings JR, Ferrell RE, Manuck SB (2005). Heart rate variability is associated with polymorphic variation in the choline transporter gene.. Psychosom Med.

[10] Matsunaga T, Gu N, Yamazaki H, Tsuda M, Adachi T (2009). Association of UCP2 and UCP3 polymorphisms with heart rate variability in Japanese men.. J Hypertens.

[11] Matsunaga T, Yasuda K, Adachi T, Gu N, Yamamura T (2007). Association of beta-adrenoceptor polymorphisms with cardiac autonomic modulation in Japanese males.. Am Heart J.

[12] Yang AC, Chen TJ, Tsai SJ, Hong CJ, Kuo CH (2010). BDNF Val66Met polymorphism alters sympathovagal balance in healthy subjects.. American Journal of Medical Genetics B.

[13] Cheng D, Tsai SJ, Hong CJ, Yang AC (2009). Reduced physiological complexity in robust elderly adults with the APOE epsilon4 allele.. PLoS One.

[14] Ranade K, Jorgenson E, Sheu WH, Pei D, Hsiung CA (2002). A polymorphism in the beta1 adrenergic receptor is associated with resting heart rate.. Am J Hum Genet.

[15] Wilk JB, Myers RH, Pankow JS, Hunt SC, Leppert MF (2006). Adrenergic receptor polymorphisms associated with resting heart rate: the HyperGEN Study.. Ann Hum Genet.

[16] Peng CK, Yang AC, Goldberger AL (2007). Statistical physics approach to categorize biologic signals: from heart rate dynamics to DNA sequences.. Chaos.

[17] Yang AC, Hseu SS, Yien HW, Goldberger AL, Peng CK (2003). Linguistic analysis of the human heartbeat using frequency and rank order statistics.. Phys Rev Lett.

[18] Goldberger AL, Amaral LA, Glass L, Hausdorff JM, Ivanov PC (2000). PhysioBank, PhysioToolkit, and PhysioNet: components of a new research resource for complex physiologic signals.. Circulation.

[19] Task-Force (1996). Task Force of the European Society of Cardiology and the North American Society of Pacing and Electrophysiology: Heart rate variability: standards of measurement, physiological interpretation and clinical use.. Circulation.

[20] Mietus JE, Peng CK, Henry I, Goldsmith RL, Goldberger AL (2002). The pNNx files: re-examining a widely used heart rate variability measure.. Heart.

[21] Goldberger JJ, Challapalli S, Tung R, Parker MA, Kadish AH (2001). Relationship of heart rate variability to parasympathetic effect.. Circulation.

[22] Katona PG, Jih F (1975). Respiratory sinus arrhythmia: noninvasive measure of parasympathetic cardiac control.. J Appl Physiol.

[23] Pomeranz B, Macaulay RJ, Caudill MA, Kutz I, Adam D (1985). Assessment of autonomic function in humans by heart rate spectral analysis.. Am J Physiol.

[24] Malliani A, Lombardi F, Pagani M (1994). Power spectrum analysis of heart rate variability: a tool to explore neural regulatory mechanisms.. Br Heart J.

[25] Taylor JA, Carr DL, Myers CW, Eckberg DL (1998). Mechanisms underlying very-low-frequency RR-interval oscillations in humans.. Circulation.

[26] Akselrod S, Gordon D, Ubel FA, Shannon DC, Berger AC (1981). Power spectrum analysis of heart rate fluctuation: a quantitative probe of beat-to-beat cardiovascular control.. Science.

[27] Peng CK, Havlin S, Stanley HE, Goldberger AL (1995). Quantification of scaling exponents and crossover phenomena in nonstationary heartbeat time series.. Chaos.

[28] Peng CK, Buldyrev SV, Goldberger AL, Havlin S, Sciortino F (1992). Long-range correlations in nucleotide sequences.. Nature.

[29] Costa M, Goldberger AL, Peng CK (2002). Multiscale entropy analysis of complex physiologic time series.. Phys Rev Lett.

[30] Makikallio TH, Hoiber S, Kober L, Torp-Pedersen C, Peng CK (1999). Fractal analysis of heart rate dynamics as a predictor of mortality in patients with depressed left ventricular function after acute myocardial infarction. TRACE Investigators. TRAndolapril Cardiac Evaluation.. Am J Cardiol.

[31] Makikallio TH, Seppanen T, Airaksinen KE, Koistinen J, Tulppo MP (1997). Dynamic analysis of heart rate may predict subsequent ventricular tachycardia after myocardial infarction.. Am J Cardiol.

[32] Ho KK, Moody GB, Peng CK, Mietus JE, Larson MG (1997). Predicting survival in heart failure case and control subjects by use of fully automated methods for deriving nonlinear and conventional indices of heart rate dynamics.. Circulation.

[33] Tapanainen JM, Thomsen PE, Kober L, Torp-Pedersen C, Makikallio TH (2002). Fractal analysis of heart rate variability and mortality after an acute myocardial infarction.. Am J Cardiol.

[34] Tibby SM, Frndova H, Durward A, Cox PN (2003). Novel method to quantify loss of heart rate variability in pediatric multiple organ failure.. Crit Care Med.

[35] Huikuri HV, Makikallio TH, Peng CK, Goldberger AL, Hintze U (2000). Fractal correlation properties of R-R interval dynamics and mortality in patients with depressed left ventricular function after an acute myocardial infarction.. Circulation.

[36] Ho YL, Lin C, Lin YH, Lo MT (2011). The prognostic value of non-linear analysis of heart rate variability in patients with congestive heart failure- A pilot study of multiscale entropy.. PLoS ONE.

[37] Guzzetti S, Borroni E, Garbelli PE, Ceriani E, Della Bella P (2005). Symbolic dynamics of heart rate variability: a probe to investigate cardiac autonomic modulation.. Circulation.

[38] Porta A, Guzzetti S, Montano N, Furlan R, Pagani M (2001). Entropy, entropy rate, and pattern classification as tools to typify complexity in short heart period variability series.. IEEE Trans Biomed Eng.

[39] Kurths J, Voss A, Saparin P, Witt A, Kleiner HJ (1995). Quantitative analysis of heart rate variability.. Chaos.

[40] Wessel N, Ziehmann C, Kurths J, Meyerfeldt U, Schirdewan A (2000). Short-term forecasting of life-threatening cardiac arrhythmias based on symbolic dynamics and finite-time growth rates.. Phys Rev E Stat Phys Plasmas Fluids Relat Interdiscip Topics.

[41] Yeragani VK, Nadella R, Hinze B, Yeragani S, Jampala VC (2000). Nonlinear measures of heart period variability: decreased measures of symbolic dynamics in patients with panic disorder.. Depress Anxiety.

[42] Yang AC, Peng CK, Yien HW, Goldberger AL (2003). Information categorization approach to literary authorship disputes.. Physica A.

[43] Yang AC, Goldberger AL, Peng CK (2005). Genomic classification using an information-based similarity index: application to the SARS coronavirus.. J Comput Biol.

[44] Shannon CE (1948). A mathematical theory of communication.. Bell Sys Tech J.

[45] Maestri R, Pinna GD, Allegrini P, Balocchi R, Casaleggio A (2005). Linear and non-linear indices of heart rate variability in chronic heart failure: mutual interrelationships and prognostic value.. Comput Cardiol.

[46] Yeh RG, Han YY, Shieh JS, Wang SC, Fu YC (2007). Nonrandomness index applied for heart rate variability in surgical intensive care units using frequency and rank order statistics.. Biomed Eng.

[47] Maestri R, Pinna GD, Accardo A, Allegrini P, Balocchi R (2007). Nonlinear indices of heart rate variability in chronic heart failure patients: redundancy and comparative clinical value.. J Cardiovasc Electrophysiol.

[48] Bengtsson K, Melander O, Orho-Melander M, Lindblad U, Ranstam J (2001). Polymorphism in the beta(1)-adrenergic receptor gene and hypertension.. Circulation.

[49] Humma LM, Puckett BJ, Richardson HE, Terra SG, Andrisin TE (2001). Effects of beta1-adrenoceptor genetic polymorphisms on resting hemodynamics in patients undergoing diagnostic testing for ischemia.. Am J Cardiol.

[50] Grandy SA, Denovan-Wright EM, Ferrier GR, Howlett SE (2004). Overexpression of human beta2-adrenergic receptors increases gain of excitation-contraction coupling in mouse ventricular myocytes.. Am J Physiol Heart Circ Physiol.

[51] Lohse MJ (2004). Beta-adrenoceptor polymorphisms and heart failure.. Trends Mol Med.

[52] Bleecker ER, Postma DS, Lawrance RM, Meyers DA, Ambrose HJ (2007). Effect of ADRB2 polymorphisms on response to longacting beta2-agonist therapy: a pharmacogenetic analysis of two randomised studies.. Lancet.

[53] Snyder EM, Beck KC, Dietz NM, Eisenach JH, Joyner MJ (2006). Arg16Gly polymorphism of the beta2-adrenergic receptor is associated with differences in cardiovascular function at rest and during exercise in humans.. J Physiol.

[54] Masuo K, Katsuya T, Fu Y, Rakugi H, Ogihara T (2005). Beta2-adrenoceptor polymorphisms relate to insulin resistance and sympathetic overactivity as early markers of metabolic disease in nonobese, normotensive individuals.. Am J Hypertens.

[55] Masuo K, Katsuya T, Fu Y, Rakugi H, Ogihara T (2005). Beta2- and beta3-adrenergic receptor polymorphisms are related to the onset of weight gain and blood pressure elevation over 5 years.. Circulation.

[56] Dishy V, Sofowora GG, Xie HG, Kim RB, Byrne DW (2001). The effect of common polymorphisms of the beta2-adrenergic receptor on agonist-mediated vascular desensitization.. N Engl J Med.

[57] Antelmi I, de Paula RS, Shinzato AR, Peres CA, Mansur AJ (2004). Influence of age, gender, body mass index, and functional capacity on heart rate variability in a cohort of subjects without heart disease.. Am J Cardiol.

[58] Tien YJ, Lee YS, Wu HM, Chen CH (2008). Methods for simultaneously identifying coherent local clusters with smooth global patterns in gene expression profiles.. BMC Bioinformatics.

