# Supplementary material for: Clustering Heart Rate Dynamics Is Associated with β-Adrenergic Receptor Polymorphisms: Analysis by Information-Based Similarity Index
Source: PLoS One. 2011 May 4;6(5):e19232. doi: 10.1371/journal.pone.0019232 (PMC3087751; doi:10.1371/journal.pone.0019232)
Supplement: Table S1 — Demographic data according to β1-adrenergic receptor Ser49Gly genotype. (DOC) [file pone.0019232.s001.doc]

**Table S1.** Demographic data according to β1-adrenergic receptor Ser49Gly genotype.

| Characteristics | Ser/Ser  n = 155 | Gly allele  n = 60 | *t or χ^2^* | *p* |
| --- | --- | --- | --- | --- |
| Age, years | 33.8 ± 10.8 | 32.6 ± 10.2 | 0.769 | 0.443 |
| Gender, M/F | 42/113 | 16/44 | 0.010 | 0.920 |
| Current smoker, n | 2 | 0 | 0.010 | 0.920 |
| Body mass index, kg/m^2^ | 22.2 ± 3.9 | 22.0 ± 3.6 | 0.300 | 0.764 |

Failure in genotyping for β1-AR Ser49Gly polymorphism was noted in 6 cases.
