# Supplementary material for: Clustering Heart Rate Dynamics Is Associated with β-Adrenergic Receptor Polymorphisms: Analysis by Information-Based Similarity Index
Source: PLoS One. 2011 May 4;6(5):e19232. doi: 10.1371/journal.pone.0019232 (PMC3087751; doi:10.1371/journal.pone.0019232)
Supplement: Table S2 — Demographic data according to β2-adrenergic receptor Arg16Gly and Gln27Glu genotype. (DOC) [file pone.0019232.s002.doc]

**Table S2.** Demographic data according to β2-adrenergic receptor Arg16Gly and Gln27Glu genotype.

| Characteristics | β2-AR Arg16Gly genotype | | | *F or χ^2^* | *P* | β2-AR Gln27Glu genotype | | *t or χ^2^* | *P* |
| --- | --- | --- | --- | --- | --- | --- | --- | --- | --- |
|  | Arg16/Arg16  n = 78 | Arg16/Gly16  N = 101 | Gly16/Gly16  n = 42 |  |  | Gln/Gln  n = 181 | Glu allele  n = 40 |  |  |
| Age, years | 35.3 ± 12.0 | 32.9 ± 10.1 | 32.2 ± 9.9 | 1.536 | 0.217 | 34.0 ± 11.0 | 32.1 ± 10.0 | 1.012 | 0.313 |
| Gender, M/F | 22/56 | 27/74 | 10/32 | 0.270 | 0.874 | 51/130 | 8/32 | 0.740 | 0.390 |
| Current smoker, n | 0 | 2 | 0 | 2.400 | 0.301 | 2 | 0 | 0.060 | 0.806 |
| Body mass index, kg/m^2^ | 22.2 ± 3.8 | 22.9 ± 3.9 | 21.4 ± 3.4 | 0.402 | 0.670 | 22.1 ± 3.6 | 22.1 ± 4.5 | 0.001 | 0.999 |
