# Supplementary material for: Clustering Heart Rate Dynamics Is Associated with β-Adrenergic Receptor Polymorphisms: Analysis by Information-Based Similarity Index
Source: PLoS One. 2011 May 4;6(5):e19232. doi: 10.1371/journal.pone.0019232 (PMC3087751; doi:10.1371/journal.pone.0019232)
Supplement: Table S3 — Partial correlations between non-randomness index and heart rate variability components. (DOC) [file pone.0019232.s003.doc]

**Table S3.** Partial correlations between non-randomness index and heart rate variability components.

| Relationship with HRV indices | r (n=221) | *p* value |
| --- | --- | --- |
| Mean heart rate, beats/minute | -0.070 | 0.299 |
| SDNN, ms | -0.064 | 0.345 |
| RMSSD, ms | -0.274 | <0.001 |
| pNN50, % | -0.279 | <0.001 |
| VLF power, ln(ms^2^/Hz) | -0.021 | -0.021 |
| LF power, ln(ms^2^/Hz) | 0.185 | 0.185 |
| HF power, ln(ms^2^/Hz) | -0.049 | 0.474 |
| LF/HF ratio, normalized units | 0.234 | 0.001 |

r: partial correlation coefficient, controlling for age and body mass index.

Non-randomness measure was determined by estimating the information-based similarity index between raw interbeat interval time series and randomly shuffled surrogates (see Methods).

SDNN: standard deviation of the normal interbeat intervals

RMSSD: the root mean square successive difference between adjacent normal interbeat intervals

pNN50: percentage of adjacent intervals that varied by greater than 50 ms

VLF: very low-frequency component of heart rate variability (0.003–0.04 Hz)

LF: low-frequency component of heart rate variability (0.04–0.15 Hz)

HF: high-frequency component of heart rate variability (0.15–0.4 Hz)

LF/HF: low-frequency:high-frequency ratio
