# Supplementary material for: Clustering Heart Rate Dynamics Is Associated with β-Adrenergic Receptor Polymorphisms: Analysis by Information-Based Similarity Index
Source: PLoS One. 2011 May 4;6(5):e19232. doi: 10.1371/journal.pone.0019232 (PMC3087751; doi:10.1371/journal.pone.0019232)
Supplement: Table S4 — Genotype and standard heart rate variability characteristics according to two major clusters by a generalized association plot. (DOC) [file pone.0019232.s004.doc]

**Table S4.** Genotype and standard heart rate variability characteristics according to two major clusters by a generalized association plot

| First half ECG data |  |  |  |
| --- | --- | --- | --- |
|  | Cluster 1  n = 132 | Cluster 2  n = 89 | *p* |
| β1-AR Ser49Gly, n (Ser/Ser vs. Gly allele)* | 32/96 | 24/63 | 0.791 |
| β2-AR Arg16Gly, n (Arg/Arg vs. Gly allele) | 56/76 | 22/67 | 0.010 |
| β2-AR Gln27Glu, n (Gln/Gln vs. Gln/Glu) | 107/25 | 74/15 | 0.823 |
|  |  |  |  |
| Second half ECG data |  |  |  |
|  | Cluster 1  n = 145 | Cluster 2  n = 76 | *p* |
| β1-AR Ser49Gly, n (Ser/Ser vs. Gly allele)* | 35/106 | 25/49 | 0.218 |
| β2-AR Arg16Gly, n (Arg/Arg vs. Gly allele) | 62/83 | 18/58 | 0.008 |
| β2-AR Gln27Glu, n (Gln/Gln vs. Gln/Glu) | 121/24 | 60/16 | 0.522 |

AR: adrenergic receptor

* Failure in genotyping for β1-AR Ser49Gly polymorphism was noted in 6 cases.
